# Transnational voting as collective action in liminal occasion: Examination of political mobilization of non‐resident Turkish citizens in Germany

**DOI:** 10.1111/bjso.70152

**Published:** 2026-11-06

**Authors:** Meral Gezici Yalçın, Elif Sandal‐Önal

**Affiliations:** ^1^ Institute for Interdisciplinary Research on Conflict and Violence Bielefeld University Bielefeld Germany

**Keywords:** collective action, contested citizenship, dual exclusion, liminality, migrants, transnational voting

## Abstract

This study examines transnational voting as collective action in a liminal occasion. Focusing on Turkey‐origin residents in Germany during and after the 2023 Turkish national elections, it combines multi‐sited ethnographic fieldwork with 17 post‐election interviews. The ethnography shows how party alliances transformed formal voting rights into mobilization through organizational infrastructures, trusted networks, symbolic venues and ideological repertoires. The interviews show how these mobilizations were interpreted through contested entitlement, anti‐immigrant sentiment, protective transnationalism and in‐betweenness. Together, the findings show that transnational voting becomes collective action when it is organized through shared infrastructures, activated through social ties and interpreted as a claim to rights, recognition, protection or opposition. The article contributes to research on collective action, diaspora politics and transnational citizenship by showing how migrants' agency emerges from dual inclusion and exclusion across national contexts.

## INTRODUCTION

Transnational voting—the exercise of electoral rights in a country of origin while residing abroad—has become a prominent yet contested feature of contemporary citizenship. More than a legal mechanism of enfranchisement, it reveals how migrants navigate multiple political communities and forms of belonging, thereby blurring the boundaries between the political spheres of origin and residence (Mügge et al., 2021). Public debates frequently question whether citizens living abroad should influence decisions that primarily affect residents of the country of origin (Bauböck, 2016; Burgess, 2020; Caramani & Grotz, 2016b; Lafleur, 2013; Lopez‐Guerra, 2005; Østergaard‐Nielsen et al., 2019; Whitaker & Wellman, 2024). Such controversies become particularly pronounced when non‐resident citizens are also racialized, minoritized or politically divided in their countries of settlement (El‐Tayeb, 2016; Lentin, 2015; Moffitt et al., 2019). In these circumstances, transnational voting is not simply an electoral procedure but a liminal occasion: a transitional moment in which established boundaries are temporarily unsettled, identities renegotiated and alternative political orders imagined through collective meaning‐making. Drawing on Stenner's (2017) transdisciplinary approach to psychosocial, we conceptualize transnational voting as a liminal occasion.

### Voting as liminal occasion

Stenner (2017) understands liminality as a process of emergence or becoming through which new processes, forms, structures, experiences and entities come into being (see also Thomassen, 2014; van Gennep, 1960 [1909]). The liminal present is an intermediate zone that connects continuity with transformation, remaining tied to the old order while anticipating a new one. The future is therefore imagined by drawing the past—often in alternatively charged ways—into the present. It marks a passage between an ‘old normal’ and a possible ‘new normal’, often guided by ‘master of ceremonies’—in electoral contexts, we can think of party leaders, campaign organizers or election officials.

According to Stenner (2017), rituals and ceremonies bring different temporalities together through what he describes as devised or staged liminality: past experiences remain present through memory, while anticipated futures shape the meaning of present action. We argue that elections display a similar ritual structure through activities such as appointing election officials, monitoring by party observers, issuing proof‐of‐residence certificates, organizing rallies, delivering political speeches and conducting campaigns in public spaces. In this sense, temporal flows intersect through fabulation, which is mediated by symbolic practices and cultural forms that organize emotions, experiences and transitions thereby making an emerging political reality imaginable (Stenner, 2017). In the context of elections, media narratives, political myths, music, flags, slogans and anthems can be understood as forms of such fabulation, connecting particular events to broader collective histories and anticipated futures. Through these performances and activities, Stenner (2017) argues, people acquire political meaning, contributing to processes of cultural self‐formation by enabling participants to imagine who they are—and who they might become—as both individuals and members of a political collective.

We argue that transnational voting brings together two temporalities: the relatively short and cyclical temporality of an election and the more prolonged, potentially liminal experience of migration (for permanent liminality, see Gezici Yalçın & Yalçın, 2026). Their intersection turns voting into a moment in which memories of origin, experiences of settlement and imagined political futures are brought into a shared present.

### Migration and citizenship at thresholds

Migrants are in an enduring position between the political communities of settlement and origin, even long after the formal settlement (Bendixsen & Eriksen, 2018; Hartonen et al., 2022; Sulaiman‐Hill & Thompson, 2012). Migrants may acquire rights while remaining socially marked as outsiders in the settlement country through membership categories such as ‘people with migration background’, ‘labour migrant’ or ‘refugee’ (e.g. Ruhs, 2013; Xiang & Lindquist, 2014) and stigmatized as insufficiently integrated (Bauböck, 2016; Lentin, 2015; Lopez‐Guerra, 2005; Moffitt et al., 2019; Owen, 2011). At the same time, origin countries may address them as national subjects while questioning their political legitimacy and loyalty because they reside elsewhere (Aydın, 2016; Baser & Féron, 2022; El Masri, 2020; Favell, 2022; Kaya, 2019; Şahin‐Mencütek & Baser, 2017; Sandal‐Önal et al., 2022; Sayad, 2004). Experiences of dual denial (Cárdenas et al., 2021), or a sense of remaining permanently ‘alien’ in both nation‐states (Favell, 2022), may intensify suspicions of insufficient belonging (Çelik, 2015; Hall, 2021; Harpaz & Mateos, 2018). The liminal position of ‘no longer’ and ‘not yet’ (see Stenner, 2017; Szakolczai, 2015), becomes especially fraught in contemporary ‘nativist times’, when dominant political movements challenge fundamental rights, minority protections and constitutional principles (Hurenkamp & de Waal, 2026). Anti‐immigrant sentiments in the country of residence and non‐resident delegitimization in the country of origin, therefore operate together producing a condition of dual exclusion that liminality theory helps to conceptualize (see Figure 1).

**FIGURE 1:**
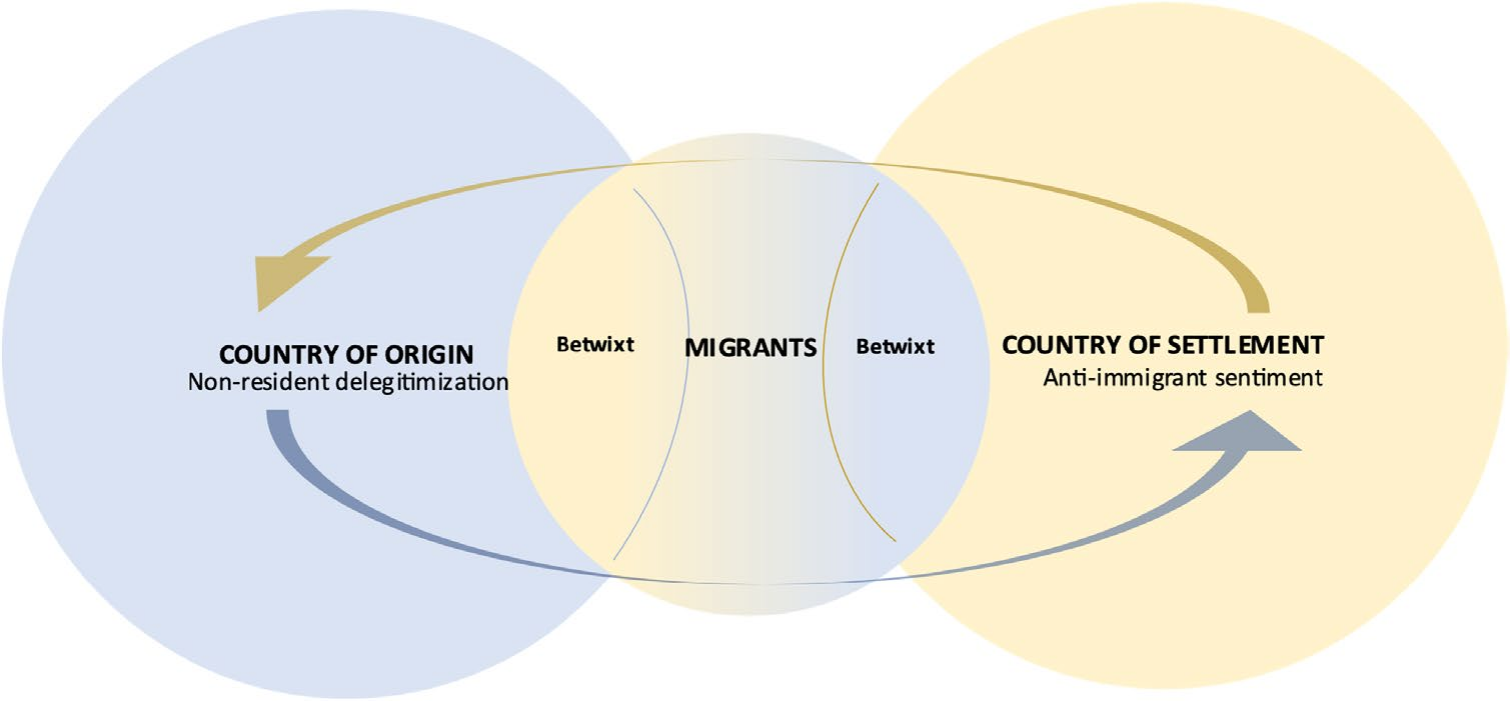
Dual exclusion across transnational space. This figure illustrates the threshold position created when exclusionary pressures in settlement and origin contexts operate simultaneously. This does not imply that all migrants hold dual citizenship or voting rights in both countries. Citizenship status and political rights vary substantially.

Migration is, we argue, *a liminal occasion* (cf. Stenner, 2017), insofar as it entails processes of psychological undoing and becoming at volatile thresholds. As Stenner argues, liminality is both relational and processual. We suggest that migration is likewise relational, because its meaning derives from connections to other people and events and processual, because the present is shaped by a past that gives rise to imagined futures. Migrants' agency is therefore situated and relational. Agency is the individual and collective capacity to interpret one's position and act through available networks under unequal conditions (Häkli & Kallio, 2014, see also Bandura, 2006), and shaped by belonging, recognition, emotion and collective continuity (Hage, 2021; Obradović, 2019; Sani et al., 2008; Smeekes & Verkuyten, 2015). Migrants exercise agency within such relations by interpreting their position, creating networks, making claims and participating politically across borders (Favell, 2022; Klandermans et al., 2008; Moghaddam & Perreault, 1992). Their capacity to act depends on institutional opportunities, family and kinship ties, political organizations, trusted intermediaries, digital communication and material resources such as information, transport and access to campaign events (Bilecen, 2021; Bilecen & Lubbers, 2021; Faist & Özveren, 2004; Lacroix et al., 2016; Ryan, 2023).

When migrants' agency is denied, exclusion may evoke grievance, anxiety, nostalgia or resentment (Düzen et al., 2022; Smeekes & Verkuyten, 2013). Migrant agency also unfolds within competing state projects. Origin‐state actors and political organizations can mobilize these emotions by addressing migrants as members of a valued, endangered or historically continuous collectivity (Sandal‐Önal et al., 2022). Shared grievances and attachments may then crystalize into a politicized collective identity that connects identification, perceived efficacy and action (Simon & Klandermans, 2001; van Zomeren et al., 2008). The same liminal position can nevertheless generate divergent responses, depending on how exclusion is interpreted, which groups become salient and which forms of action are organizationally available. Experiences of in‐betweenness also vary by generation, class, ethnicity, religion, political orientation and encounters with racism and legal or social precarity (Ellermann, 2019; El‐Tayeb, 2016; Lentin, 2015; Moffitt et al., 2019). Citizenship may take the form of either/or through single citizenship, both through dual citizenship or neither/nor for people without recognized status (e.g. undocumented migrants). Dual citizenship may function as a strategic resource for some and as a source of suspicion or divided attachment for others (Faist & Kivisto, 2007; Favell, 2022). Liminality is therefore used here not as a totalizing description of migrant experience but as an analytic lens linking macro‐level structures of unequal inclusion, meso‐level infrastructures of mobilization and micro‐level meanings attached to transnational voting (for levels within liminality see Thomassen, 2014). Rather than presuming a homogeneous migrant public, we therefore examine how organizations, associations and interpersonal networks mobilize different segments of migrant populations. Before this fragmentation, in the following, we argue why we see transnational voting as collective action.

### Transnational voting as collective action

Research in social and political science commonly approaches transnational voting primarily as an institutional or behavioural outcome, asking who votes, under what conditions and with what consequences. Drawing on the social psychology of collective action, however, we instead conceptualize it as collective action in liminal occasion: a social change process concerned with how people experience and respond to transition at both individual and collective levels, connecting the micro‐ and macro‐level dynamics from the middle (Thomassen, 2014).

Collective action comprises practices undertaken on behalf of a group or in pursuit of shared interests, identities or moral commitments (Klandermans, 1997; Reicher, 2004; Stürmer & Simon, 2004; van Zomeren et al., 2008). Although often associated with protest and social movements, it also includes asserting rights, expressing solidarity, defending group interests and seeking political change (Bou Zeineddine & Leach, 2021; Leach & Livingstone, 2015; Sweetman et al., 2013). Voting becomes collective action when it is understood not simply as an individual choice, but as a group‐oriented act connected to shared identities, interests, grievances or moral commitments (Harb, 2024; Pettigrew, 1972; van Zomeren et al., 2017; Wagoner et al., 2021; Wright, 2001).

Transnational voting takes place in a contested field where states extend electoral rights across borders while simultaneously regulating and politicizing the legitimacy of citizens abroad (Bauböck, 2016; Burgess, 2020; Caramani & Grotz, 2016b; Lafleur, 2013). Research has examined normative foundations, turnout, party outreach and institutional arrangements (Bauböck, 2016; Burgess, 2020; Caramani & Grotz, 2016a, 2016b; Lafleur, 2013; Lopez‐Guerra, 2005; Whitaker & Wellman, 2024). This scholarship shows that voting from abroad is not merely a technical extension of suffrage but also a site of struggle over membership, belonging and the boundaries of the demos. Migrants may consequently be politically marginalized yet symbolically instrumentalized—welcomed when useful and questioned when inconvenient (Abadan‐Unat, 2002; Kahn, 2024; Kastoryano, 2002; Østergaard‐Nielsen, 2003; Owen, 2009; Sayad, 2004; Soysal, 1994).

Participation from abroad is shaped by electoral arrangements, party outreach, migrant organizations, settlement‐country opportunity structures and attachments to both origin and settlement contexts (Ciornei & Østergaard‐Nielsen, 2020; Goldberg & Lanz, 2021; Sevi et al., 2020; van Haute & Kernalegenn, 2021; Whitaker & Wellman, 2024). Following Klandermans (1997, 2004), voting becomes collective action when it is perceived as consequential, enacted through a meaningful group identity and situated within broader ideological and moral narratives. Party organizations, migrant associations and interpersonal networks create these conditions by reducing participation costs, recruiting voters through trusted ties and framing participation as loyalty, democratic duty, resistance, representation or the defence of collective rights.

The collective interests invoked through transnational voting cannot be assumed in advance; they are socially constructed and contested through discourse and mobilization (see Reicher & Sani, 1998; Sani & Reicher, 1999). Voting may be framed as defending the homeland, restoring democracy, resisting authoritarianism, protecting marginalized communities or asserting migrants' continued presence within the political community. Its collective dimension lies not in casting a ballot, but in the organizational, relational and meaning‐making processes that connect participation to a wider collectivity.

Elections are institutionalized and cyclical, governed by predefined rules and enacted through campaigns, transport to polling stations, interpersonal recruitment, digital coordination and symbolic performances. These practices can be understood as examples of what Stenner (2017) terms devised liminality. Voting may also be supplemented—or, where access is constrained, substituted—by non‐institutionalized political action (Klandermans, 1997; van Stekelenburg et al., 2016). During election periods, state‐organized rituals and campaign practices turn individual experiences into a shared liminal process. They temporarily suspend ordinary routines, stage movement from an established order towards a possible future and create occasions on which destabilized identities are reaffirmed or challenged (see Obradović et al., 2025 for Queen Elizabeth's II death). By examining meaning‐making within liminal occasions of voting we can identify and analyse the political and national identity politics that ingroup members seek to mobilize for the collective future reflects itself in particular agendas of social actors towards the future of the nation (see Reicher & Hopkins, 2001).

This liminal occasion is organized through narratives of belonging, loyalty, resistance and democratic responsibility. It activates group boundaries by raising the question of who belongs to ‘us’ and links present action to competing interpretations of the national past and future. In the Turkish context, for example, campaigns may contrast the republic's ‘second century’, emphasizing continuity with the order founded by Atatürk, with a ‘new century’ associated with Erdoğan (Uluşahin et al., 2026). In authoritarian settings, where elections may be treated as the only legitimate channel for expressing grievances, participation itself can be framed as resistance and voting as protest (Otjes et al., 2020; Uluğ et al., 2025). A liminality perspective therefore shifts attention from turnout as an aggregate outcome to the processes through which voting abroad becomes a collectively organized and symbolically meaningful practice for negotiating rights, recognition, exclusion and political agency across borders.

## RESEARCH CONTEXT: VOTING IN TURKEY FROM GERMANY

Migrants from Turkey and their descendants constitute one of Germany's largest and most politically visible migrant‐origin populations. They have long occupied a contested position: economically, socially and symbolically significant to both Germany and Turkey, yet often treated as not fully belonging in either context (e.g. Abadan‐Unat, 2002; Kastoryano, 2002; Kaya, 2013; Mandel, 2008; Schönwälder, 2006; Soysal, 1994). Labels such as *Ausländer* in Germany and *Almancı* (*Deutschländer*) in Turkey index this recurring experience of in‐betweenness (e.g. Kahn, 2020).

Turkey introduced external voting through a constitutional reform in 1995, but voting from abroad remained difficult for many years, partly because Germany did not permit Turkish electoral procedures on its territory. It became practicable only after further reforms in Turkey and a change in Germany's position following a 2012 ruling by the European Court of Human Rights (Şahin‐Mencütek & Erdoğan, 2016; Sevi et al., 2020). Turkish national elections have since generated intense party competition, associational mobilization and public controversy in Germany (Arkilic, 2021; Becker‐Bertau, 2020; Şahin‐Mencütek & Baser, 2017).

This external voting field is organizationally uneven. Party‐ and state‐aligned organizations, mosque networks, local associations, consular ties and digital channels translate formal voting rights into mobilization (Becker‐Bertau, 2020; Şahin‐Mencütek & Baser, 2017; van Haute & Kernalegenn, 2021). Pro‐government mobilization, particularly by the Justice and Development Party (AKP), has been linked to the Union of International Democrats (UID), Turkish‐Islamic Union for Religious Affairs (DITIB) mosques and consular networks; opposition and Kurdish‐left alliances have likewise mobilized supporters abroad (see Arkilic, 2021; Baser, 2017; Kaya, 2019). These organizational dynamics intersect with divisions of citizenship, generation, ethnicity, religion, class, gender, migration history and political orientation.

Public debate in Germany nevertheless often portrays the Turkey‐origin electorate as a homogeneous pro‐Erdoğan bloc (von Schwerin, 2023). The 2023 election figures complicate this image. Approximately 1.5 million Turkish citizens in Germany were eligible to vote; turnout was 48.7%, and 67% of those who supported Erdoğan. His voters thus represented roughly one third of the eligible electorate and a still smaller proportion of the wider Turkey‐origin population, which also includes non‐voters, opposition supporters, German citizens without Turkish voting rights, dual citizens and politically ambivalent individuals.

The case of Turkey‐origin residents in Germany during and after the 2023 Turkish national elections is therefore analytically productive. It combines a large and internally heterogeneous population, a highly visible system of transnational voting, dense party‐linked and associational infrastructures and recurring controversies in both countries over loyalty, integration and support for Erdoğan (Arkilic, 2021; Becker‐Bertau, 2020; Kaya, 2019; Şahin‐Mencütek & Baser, 2017; Sevi et al., 2020). Turkey‐origin residents may be claimed by the Turkish state as loyal nationals abroad while being treated as insufficiently integrated in Germany (Kahn, 2024; Kastoryano, 2002; Mandel, 2008). This simultaneous inclusion and exclusion create conditions associated with group‐based mobilization: perceived threat, contested group boundaries and competing constructions of collective interests (Klandermans et al., 2008; Simon & Klandermans, 2001; van Zomeren et al., 2008). Yet responses range from intense mobilization to opposition, ambivalence, non‐participation or exclusion, underscoring that Turkey‐origin residents do not form a homogeneous political bloc.

To capture this complexity, the article integrates liminality and collective action. Liminality illuminates how voters and non‐voters navigate through a politically mediated threshold between origin and settlement contexts and how electoral participation may redefine status, identity and imagined collective futures. Collective action theory, in turn, explains how this position becomes politically consequential through organized, networked and meaning‐laden participation. The empirical design reflects this synthesis. Study 1 examines how political alliances mobilized non‐resident Turkish citizens in Germany through organizational infrastructures, networks and symbolic repertoires. Study 2 investigates how Turkey‐origin residents interpreted voting through experiences of exclusion, entitlement, anti‐immigrant sentiment and cross‐border belonging. Together, the studies show how transnational voting becomes collective action when organizational mobilization converges with lived meaning‐making.

## RESEARCH AIM AND METHODOLOGICAL APPROACH

This study addresses two interrelated research questions. First, how do political alliances mobilize Turkey‐origin residents in Germany during 2023 Turkish national elections through instrumentality, identity and ideology? Second, how do Turkey‐origin residents—including eligible voters and people without Turkish voting rights—construct and contest group boundaries, norms and collective interests in relation to transnational voting? Rather than presuming fixed ethno‐national identifications, we examine how participants define group boundaries, evaluate the legitimacy of non‐resident voting and narrate its advantages, disadvantages and collective stakes. We further ask how these arguments draw on cross‐border experiences of inclusion and exclusion and on memories of the past and imaginations of the collective future.

The two questions connect collective action with liminality while keeping their analytical functions distinct. Collective action theory explains how political participation is organized and made consequential through identities, networks, perceived efficacy and ideological narratives. Liminality theory directs attention to the election period as a structured threshold (devised liminality): a process which established classifications and routines are temporarily unsettled, competing futures become imaginable and belonging to the political communities of origin and settlement is renegotiated. Transnational voting is therefore approached neither as the automatic expression of a stable and fixed identity nor as evidence that all migrants share the same in‐between condition. Instead, it is examined as a liminal occasion nested within the longer and unequal temporalities of migration—a politically mediated process through which dual exclusion may be reproduced, challenged or transformed into agency.

This theoretical distinction informed the empirical design. Participatory observation addresses the first research question by tracing instrumentality, identity and ideology in campaign practices, organizational infrastructures, venues, rituals and symbolic repertoires following Klandermans' (1997, 2004) tripartite framework and Stenner's (2017) liminality approach. It captured how alliances organized electoral passage: who mobilized, through which networks and spaces, with what material resources and symbols, and under which conditions of access. Particular attention was paid to the practices through which the election was staged as a consequential transition between a contested past, an unsettled present and alternative political futures.

Semi‐structured interviews addressed the second research question by examining participants' meaning‐making through the Structural Analysis of Group Arguments (SAGA) dimensions of category inclusiveness, norms and interests (Reicher & Sani, 1998; Sani & Reicher, 1999) within a liminal occasion. They explored how participants drew and contested the boundaries of the relevant ‘we’, judged who should be entitled to vote and according to which norms, and defined the individual and collective interests at stake. The interviews also captured how participants interpreted anti‐immigrant sentiment, political recognition, cross‐border belonging and the temporal tension between continuity and change. This focus makes it possible to examine whether voting, non‐voting and exclusion from voting were experienced as confirming an existing order, suspending it or opening the possibility of a different one, a liminal occasion that ties past, present and future.

Ethnography reveals the organizational and performative production of the election as a collective liminal occasion (Study 1); interviews show how this occasion was experienced, evaluated or rejected from differently positioned lives (Study 2). Read together, they connect the meso‐level organization of mobilization with micro‐level meaning‐making and show how political infrastructures and lived interpretations jointly shape whether transnational voting becomes meaningful collective action.

The two researchers who led the coding and analysis are themselves recent immigrants to Germany, a position distinct from that of the bilingual research assistants who conducted the interviews in Study 2 and who are established members of that society. This asymmetry between those who elicited the interview material and those who analysed it is itself a form of liminal positioning, and it shaped how disclosure, rapport and interpretation unfolded at different stages of the research. Bilingual Turkish‐German research assistants conducted the interviews, facilitating access while also shaping interaction and disclosure in ways that cannot be fully anticipated. We addressed these dynamics through ongoing reflexive discussions during data collection and analysis. In presenting the findings, we sought to preserve the complexity and internal variation of participants' accounts rather than treating them as illustrations of predetermined theoretical categories. This reflexive approach was particularly important because interpretations of liminality depend on how participants themselves understood continuity, disruption, belonging and possible futures.

## STUDY 1: ETHNOGRAPHY

We conducted both participatory and non‐participatory observation during the 2023 Turkish national elections in Germany, focusing on campaign activities and public events organized by three major electoral alliances: People's Alliance (PA), National Alliance (NA) and Labour and Freedom Alliance (LFA).^1^ Data were collected in April and May 2023 following the ethical approval (Approval no. 2023‐196) in three mid‐sized German cities (K., H. and M.). The cities are not identified in order to protect the anonymity of local organizations, participants and politically sensitive field sites. The material comprises 70 pages of word document including field notes, photographs, campaign brochures of election promises and social media content of a website dating back one year.

German restrictions on foreign political rallies (Talmon, 2017), displaced much mobilization into mosques, consulates, cultural events, cafés and association buildings and caused parties to take security precautions towards the outside world. These venues functioned as threshold spaces in which civic, cultural, religious and explicitly political practices overlapped. Facebook and WhatsApp extended this liminal sphere across physical distance by circulating invitations, symbols, information and affective appeals. Combining direct observation with digital ethnography allowed us to examine how the electoral occasion was staged across interconnected spaces and how participation depended on power, trust, political affiliation and access. Participant observations were missing for Ramadan iftars, consulate and mosque activities organized by PA were only open to their circles and members, which led us to follow their Facebook account where we could read the reports of the events and shared pictures. In addition, we were first accepted and then quickly excluded from the CHP Whatsapp group without any explanation, where we observed differences in terms of approaches to researchers between cities and their central election office. Getting data only through participant observation was possible by the LFA public meeting, where our researcher identity was welcomed. These caused limited observations for PA (digital data instead of real‐life conversations) and limited temporality for NA.

### Analytical approach

The ethnographic material was analysed using Situational Analysis (Clarke et al., 2016), which is well suited to complex, multi‐actor environments such as diaspora politics. Its relational attention to discourse, positionality, material elements and social worlds allowed us to examine how the election acquired the form of collective liminal occasion. Rather than treating liminality as a fixed attribute of migrants, we traced how organizational practices temporarily suspended or intensified established boundaries, guided participants through electoral passage and made competing political futures experientially present.

Three complementary mapping strategies were used. Situational map identified the human and non‐human elements of the electoral occasion—including organizations, political actors, venues, events, digital platforms, transport and symbolic practices—and traced their interrelations. Social worlds and arenas map located collective actors such as UID, DITIB, local associations and activist groups within overlapping and competing fields of action (see Table 1). Positional map charted articulated and absent positions on central political questions without reducing these positions to particular arenas. Together, these maps showed how the electoral passage was organized spatially, materially and discursively and how inclusion and exclusion were produced through differential access to venues, networks and both material and symbolic resources.

**TABLE 1:** Three maps generated from the data.

**Map 1. Situation map**	
**Dimension**	**Elements in the situation**
Human actors	Voters; young voters; older voters; Kurdish speakers; German speakers; activists; party observers; consular staff; journalists; event audiences; families; researchers
Collective actors	YSP/HDP networks; CHP local associations; AKP/UID networks; MHP observers; Kurdish political milieu; Turkish associations; religious associations; civil society networks
Institutions	Turkish consulates; YSK/electoral system; German authorities; security personnel; municipalities; media; political parties in Türkiye
Spaces	Airport; polling station; street; theatre; mosque; association venue; consulate; online space
Objects/material elements	Ballot paper; ID card; flag; leaflet; phone; WhatsApp; Facebook; political symbols
Discourses	Homeland; nation; democracy; diaspora; integration; racism; religion; family; generation; citizenship
Affects	Belonging; trust; distrust; enthusiasm; tension; solidarity; feeling at home; exclusion
Practices	Voting; campaigning; observing; dancing/halay; giving speeches; commemoration; security checks; networking
**Map 2. Social worlds/Arenas map**	
**Social world/arena**	**Actors, practices and sites**
Türkiye politics	Political parties; elections; politicians; YSK; polarization; campaign strategies
Electoral arena	Consulates; polling sites; airport voting location; ballot officials; party observers; voters; security procedures; language assistance
Party/movement arena	YSP/HDP; CHP; AKP/UID; MHP; Kurdish political activism; local campaign networks
Civil and religious arena	Turkish associations; mosques; religious associations; community events; iftar events; charity events; commemorative gatherings
Public/media arena	Panels; journalists; Facebook; WhatsApp groups; public speeches; political discussion; media framing
Everyday life arena	Family; generation; neighbourhood; migration biographies; belonging; language use; religion; racism; integration
German institutional arena	German law; local authorities; permissions; security regulations; citizenship regime; integration discourse; local public order
**Map 3. Positional map**	
**Position/axis**	**Meaning in the data**
Axis 1: Political orientation	Türkiye‐centred; Germany‐centred; transnational/dual orientation
Position A: Democratic right	Citizens retain voting rights; participation is legitimate; voting is a democratic act; political voice is not necessarily limited by place of residence
Position B: Transnational duty	Responsibility to homeland; maintaining ties to Türkiye; protecting collective identity; supporting nation/community from abroad
Position C: Problematic participation	Voting on a country where one does not live; distance from everyday consequences; questions of legitimacy; tension between residence and representation
Axis 2: Understanding of belonging	Inherited/communal; civic/chosen; mixed/negotiated
Position D: Conditionally legitimate	Legitimacy linked to being informed, maintaining ties, citizenship status, integration, generation or language competence
Position E: Belonging through family/religion/milieu	Family traditions, religion, community environment and intergenerational continuity shape political belonging and preferences
Position F: Belonging through civic integration	Education and integration matter; distance from homeland politics may increase; local participation in Germany becomes more important
Position G: Multiple belonging	Belonging to both Germany and Türkiye; bilingual/bicultural participation; transnational citizenship practices; political life spanning more than one national space

## RESULTS

All three (situational, arena and positional) maps were produced separately then organized under three dimensions of Klandermans (1997, 2004): instrumentality, identity and ideology (see Table 2). Instrumentality concerns the infrastructures that enabled, restricted and guided electoral passage into participation. Identity concerns the collective subjects called into being and the boundaries temporarily reaffirmed or reworked. Ideology concerns the moral, affective and temporal narratives through which voting was linked to an established order or an imagined alternative future. Below, results are presented specific to each alliance.

**TABLE 2:** Integrated dimensions.

Dimensions of Klandermans (1997)	Dimensions of situational maps
Instrumentality	Institutions, spaces, objects/material elements, practices, Türkiye politics, electoral arena, party/movement arena, civil and religious arena, public/media arena, German institutional arena
Identity	Human actors, collective actors, everyday life arena, axis 2: understanding of belonging, position D: conditionally legitimate, position E: belonging through family/religion/milieu, position F: belonging through civic integration
Ideology	Discourses, affects, axis 1: political orientation: understanding of belonging, position A: democratic right, position B: transnational duty, position C: problematic participation

### Instrumentality: Resources, institutions and spaces for electoral passage

Instrumentality was evident in the alliances' unequal capacity to transform formal voting rights into actual participation. PA possessed the strongest infrastructure. UID branches, DITIB mosques, consular connections and state‐aligned associations provided communication channels, venues, voter transportation and symbolic proximity to Turkish political power. Buses to polling stations (see Figure 2 picture at bottom), publicized events with Turkish politicians and campaign materials circulated through social media reduced the practical costs of participation while displaying organizational strength. These infrastructures did more than facilitate turnout: they organized participants' movement into and through the electoral occasion.

**FIGURE 2:**
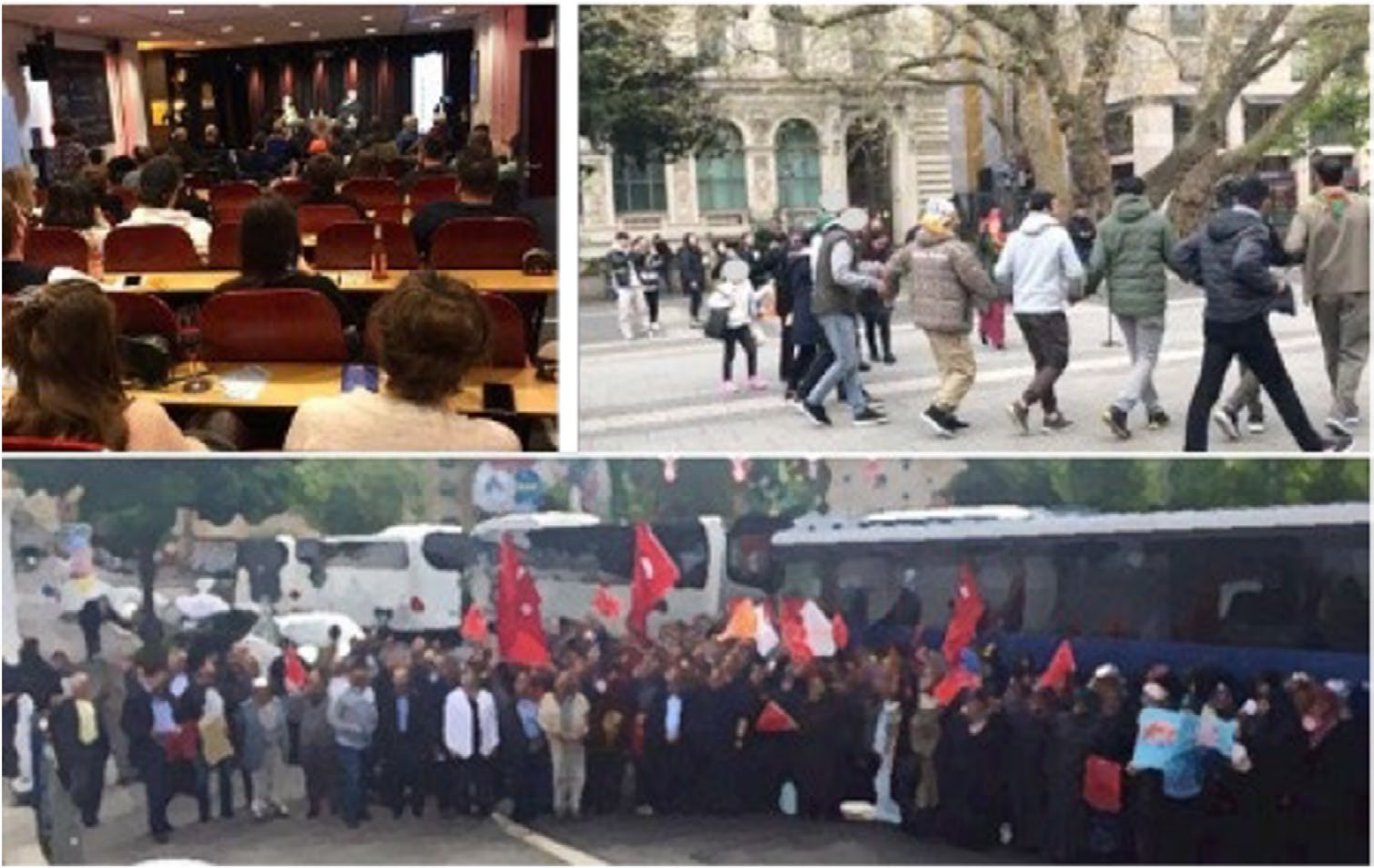
Political mobilization strategies of alliances in the public space. Upper left and upper right photographs depict a public discussion event and a demonstration organized by the LFA respectively; both photographs were taken by the research team during ethnographic fieldwork. The lower photograph, depicting voter transportation organized by the PA, was taken from their social media and anonymized for ethical reasons.

NA relied on more localized and trust‐based resources, including election information offices, civic events, interpersonal contacts and WhatsApp groups. Its capacity was less institutionalized and depended more heavily on local credibility and available resources. LFA had fewer institutional resources but converted activist and cultural infrastructures into visible collective action through demonstrations, discussion events, Kurdish cultural associations, halay dances, zılgıt ululations, slogans and cooperation with German leftist groups (see Figure 2, both above pictures). Each alliance thus constructed a different pathway into the same electoral threshold.

Access formed a central part of these pathways. Some PA‐related venues were formally public yet practically semi‐closed; researchers entered an NA WhatsApp group only briefly before being removed; and LFA events were relatively open but structurally marginalized within the wider electoral arena. Facebook and WhatsApp connected dispersed participants to campaign activities, but these digital spaces were also stratified by prior affiliation, trust and insider status. Instrumentality therefore included boundary work: alliances controlled not only resources but also who received information, who could enter mobilization spaces and whose participation appeared legitimate. Organizational actors consequently operated as guides through the liminal passage while simultaneously regulating access to it.

### Identity: Collective subjects and boundaries of belonging

The electoral occasion intensified questions of collective identity by inviting participants to locate themselves within competing versions of the political community. PA addressed diaspora members as loyal, morally responsible and religiously grounded Turkish‐Muslim actors in Europe. Mosques, Eid celebrations, martyr commemorations, consular activities and references to ‘Euro‐Turks’ or 'Muslims in Europe' positioned migrants as guardians of the homeland. This repertoire fused religious faith, national belonging and political loyalty to the Turkish state, implicitly placing those who did not share this configuration outside the authentic political community. PA thus sought to stabilize the threshold by extending an established national‐religious order across borders.

NA offered a secular‐republican repertoire centred on civic duty, Kemalist symbolism and democratic responsibility. Events such as the 23 April Children's Festival, commemorating the founding of the Turkish Grand National Assembly, connected electoral mobilization to established rituals of republican nationhood. Participants were addressed as custodians of the republic rather than members of a religious‐political project. This frame also drew boundaries: it privileged a civic‐republican version of Turkishness and presented the election as a passage through which the republic might be defended or restored.

LFA articulated a counter‐hegemonic collectivity grounded in Kurdish memory, collective grief, democratic struggle, anti‐racism and transnational solidarity. Its events addressed participants not primarily as Turkish nationals abroad, but as members of a community shaped by state repression, marginalization and solidarity with Kurdish and leftist movements in Germany and Turkey. Across three alliances, identity was therefore neither pre‐given nor merely expressed. It was performed and contested within the liminal occasion. Each repertoire defined who could be part of the relevant ‘we’, who could feel addressed in particular spaces, and which collective subject should emerge from the electoral transition.

### Ideology: Moral claims and symbolic repertoires

Ideology refers here to the moral and affective, and temporal meanings through which alliances made voting consequential. PA mobilized a national‐religious ideology that joined loyalty to Turkey, Islamic morality and geopolitical responsibility. Martyr commemorations, mosque‐based events, national symbols and appeals to sacrifice framed voting as an obligation towards the homeland, the national community and its political leadership. These practices functioned as liminal affective technologies: they generated shared emotions, connected present participation to narratives of past sacrifice, and projected continuity with the existing political order into the future. Within this moral universe, abstention or opposition could acquire the implicit meaning of betrayal.

NA framed voting as civic duty, democratic responsibility and commitment to secular‐republican citizenship. Kemalist symbols and the republican constitutional tradition anchored an imagined future in a valued political past. The election was presented as a threshold at which democratic institutions and secular citizenship could be defended or restored. Compared with PA, however, this repertoire appeared less affectively intense and less institutionally embedded. It offered a normative language of transition, but had fewer resources with which to stage it as a shared emotional experience.

LFA framed voting as resistance, counter‐memory and the defence of Kurdish rights and democratic freedoms. Although its events were often formally described as cultural gatherings—partly in response to restrictions on foreign political campaigning in Germany (Talmon, 2017)—music, dance, slogans, demonstrations and symbols of Kurdish resistance gave them political force. These practices drew histories of repression and marginalization into the electoral present while imagining a future beyond authoritarianism and the silencing of Kurdish claims. Voting was therefore situated within a wider passage of struggle rather than framed as loyalty to the homeland state.

Across alliances, ideology was performed through bodies, spaces, rituals, music, symbols and collective presence as much as through speeches or manifestos. Each alliance offered a different account of what was ending, what should endure and what might emerge after the election. Voting abroad was consequently not ideologically neutral: it became a fabulated transition in which competing moral worlds and collective futures were staged, imagined and performed.

## DISCUSSION

Study 1 shows that transnational voting became collective action through the convergence of instrumentality, identity and ideology (Klandermans, 2004; see also Wright, 2001, 2003). Alliances made participation practicable through transport, information, events, digital coordination and trusted infrastructures. At the same time, they transformed the election into a liminal occasion by reorganizing everyday spaces, intensifying group boundaries, mobilizing affect and connecting contested pasts to alternative futures. Voting thus acquired meaning not only as an individual choice but as participation in a collectively staged passage (see Stenner, 2017).

The collective interest attached to this passage was alliance‐specific. PA framed voting as homeland loyalty and continuity with a national‐religious order; NA as secular‐republican responsibility and the defence or restoration of democratic institutions; and LFA as resistance, defence of Kurdish rights and transnational solidarity. Collective action did not therefore arise from a homogeneous diaspora identity. It was produced through situated mobilization that defined the relevant ‘we’, identified what was at stake and supplied contrasting interpretations of the political transition.

The findings also reveal that liminal occasions are not necessarily open or equal spaces. Access to events, online spaces and transport networks depended on trust, affiliation and prior social ties (see Aydın, 2016; Becker‐Bertau, 2020). PA‐related spaces often operated through semi‐closed networks, LFA gatherings were more visibly open but structurally marginalized and NA occupied an ambivalent position between visibility and caution. The same infrastructures that guided some participants through the electoral passage excluded or rendered others peripheral. Liminality here did not dissolve power relations; it reorganized and made them especially visible.

The findings complicate accounts that position migrants primarily as extensions of homeland politics (see Sevi et al., 2020). Cross‐border networks enabled collective action (e.g. Bilecen, 2021; Ryan, 2023), but migrants and their organizations also actively interpreted the electoral threshold, mobilized resources, cultivated relationships and performed identities that challenged and reconfigured dominant narratives of belonging (De Genova et al., 2018; Favell, 2022; Mezzadra, 2011; Tazzioli, 2019). The election was nested within the longer liminality of migration (El Masri, 2020; Gezici Yalçın & Yalçın, 2026; Sulaiman‐Hill & Thompson, 2012; Szakolczai, 2015), yet it temporarily condensed that in‐betweenness into a structured occasion on which belonging, loyalty and collective futures became unusually explicit and politically consequential.

## STUDY 2: INTERVIEWS

Whereas Study 1 examined how political alliances organized the 2023 Turkish elections in Germany as a collective liminal occasion, Study 2 foregrounds participants' subsequent meaning‐making. The semi‐structured interviews aim to reveal how Turkey‐origin residents interpret group membership, narrate norms of legitimate participation and articulate collective interests across political contexts of Germany and Turkey. Conducted approximately 1 year after the election, they capture a post‐liminal perspective: how the electoral occasion persisted in memory, how its promises and anxieties were reassessed and whether it was understood as confirming, challenging or failing to transform participants' position. Together, the studies connect the organizational staging of transnational voting with its lived and retrospective interpretation.

### Procedure and participants

We conducted 17 semi‐structured interviews with Turkey‐origin residents in a medium‐sized German city in July and August 2024. Given the political sensitivity of the topic, a feasibility‐based recruitment strategy was adopted. Participants—10 women and seven men aged 18–64—were drawn from the closed networks and personal acquaintances of the bilingual Turkish‐German research assistants who conducted the interviews. Access to a politically sensitive topic of this kind depended on pre‐existing trust, which constrained recruitment to individuals reachable through these established relationships rather than through open or public channels. The interviewees varied in gender, age, education, migration history and citizenship status (see Table 3) and included Turkish citizens, German citizens and dual citizens, including people without Turkish voting rights. These differently positioned accounts made it possible to examine participation, non‐participation and exclusion from electoral participation rather than equating transnational voting with eligible voters alone. Participants were purposively recruited to capture variation in political positioning and experiences of transnational participation. Recruitment continued until further interviews added little to the revealed analytical patterns.

**TABLE 3:** Participant characteristics.

No.	Gender	Age	Education	Migration history	Citizenship
1	W	41	Bachelor student	Born and raised in Germany	German
2	W	29	Bachelor	Born and raised in Germany	German
3	M	30	Master	Born and raised in Germany	German
4	W	29	Bachelor	Born and raised in Germany	Dual
5	W	41	Secondary	Migrated to Germany (14 years)	Turkish
6	W	38	Bachelor	Born and raised in Germany	German
7	W	63	Secondary	Migrated to Germany (44 years)	Turkish
8	M	27	Abitur	Born and raised in Germany	Turkish
9	M	41	Bachelor	Born and raised in Germany	German
10	M	47	Abitur	Born and raised in Germany	German
11	M	55	High school	Migrated to Germany (27 years)	Turkish
12	M	64	Elementary	Migrated to Germany (50 years)	Turkish
13	W	39	High school	Migrated to Germany (20 years)	Turkish
14	M	18	Abitur	Born and raised in Germany	Dual
15	W	55	Elementary	Born and raised in Germany	Turkish
16	W	48	Secondary	Born and raised in Germany	German
17	W	54	Elementary	Migrated to Germany (30 years)	German

*Note*: Among eligible electoral participants, four supported the AKP (PA), one the CHP (NA), one the IYIP (NA), one the Green Left (LFA) and one abstained.

Interviews were conducted in German or Turkish, depending on participants' preference and lasted between 30 and 90 min. All interviews were transcribed verbatim; interviews conducted in German were subsequently translated into Turkish for analysis. Ethical approval was obtained for this study as well (Approval no. 2023‐196).

### Analytical strategy

We employed reflexive thematic analysis with a phenomenological orientation (Braun & Clarke, 2022), combining data‐driven and theory‐informed coding. Following repeated familiarization, open codes were developed, clustered into preliminary themes and refined through comparison and reflexive discussion. Both researchers independently conducted the first familiarization readings of the transcripts. The first researcher then led the coding process; the second researcher reviewed and either agreed or disagreed with the assigned codes. Disagreements were rare and were resolved through discussion. Analytic memos were kept by the first researcher throughout coding and informed these discussions. Coding proceeded inductively in the first instance; a deductive step then organized the resulting codes according to the SAGA dimensions of category inclusiveness, category norms and category interests (Reicher & Sani, 1998; Sani & Reicher, 1999). At the final stage of this process, a total of 119 codes were gathered under 16 themes and three meta‐themes informed by SAGA dimensions. Saturation was judged to have been reached once the first researcher began encountering recurring rather than new codes across successive transcripts, and the saturation criteria applied to code stability. All 17 interviews were first coded by the first researcher and subsequently reviewed in full by the second researcher, who checked that coded segments and their assigned codes were consistent between the two readings. Themes were developed and agreed jointly by both researchers.

## RESULTS

The findings move from the boundaries of the relevant ‘we’, through norms of legitimate participation, to the collective futures voting was expected to secure. This sequence shows how a liminal occasion becomes politically meaningful: it calls collective subjects into being, subjects their participation to competing rules of entitlement and links present action to hoped‐for or feared futures. Table 4 provides a summary of the thematic structure with examples of codes emerging from the reflexive thematic analysis.

**TABLE 4:** Examples of codes under relevant themes.

Meta‐themes	Themes	Example of codes
Category inclusiveness: Who are we?	Citizen categories	Turkish citizen, blue card holder, German citizen, dual citizens
	Ethno‐national categories	Turkish, German, Kurdish
	Hyphenated categories	German‐Turks, German‐Kurds
	Political categories	Patriot, voter crise, AKP youth, Erdogan supporter, Kurdish activist, CHP voter, republican, democrats, (non)eligible voter, Atatürkçü
	Place‐based categories	Turkey‐origin, Turks abroad, residents
	Cultural categories	Hybrid, heritage culture and origin
	Migration related categories	Gurbetçi, descendant of labour migrant, long‐established, newcomers, criminal migrants, majority at workplace, ties to homeland, outsider
	Religious categories	Muslim, Islam religion, mosque member
	In‐betweenness	Situational, in‐between
	Overarching categories	Human(ist)
Category norms: Who should vote?	Entitlement	Citizenship status, no right to local elections, difficulty of access to ballot box, non‐electoral participation
	Residence	Living place, place of birth, everyday knowledge, affecting others' life
	Knowledge/Interest	Knowledge of party politics, cultural knowledge, knowing way of life,
		Homely feelings, loyalty, (non)representing interest,
		apathy, free will, ambivalence
Category interests: What should voting achieve?	Past	Memory of past violence, discrimination, anti‐Muslim politics, exclusion, racism
	Present	Contribution to homeland, support for Palestinians, collective strength, protect rights, insecurity feeling, performance of party, cross‐border mobility, fears, a party giving hope, seeking change
	Future	Reducing migrant population, future of children, future of origin country, equality, justice, returning homeland, wait‐and‐see AfD politics

### Category inclusiveness: Who ‘we’ are across the threshold

Participants defined themselves in multiple ways—as Turkish, German, Kurdish, Muslim, German‐Turkish, German‐Kurdish or human. Others described identity as situational, embodied or spatially divided. These accounts include spatial liminality referring to the countries (I.7: ‘Most Turks have one foot in Turkey and one foot here (Germany), so we cannot break away from here, nor there’.), fluidity in a way that belonging shifts across encounters and locations (I.8: ‘I feel German among Turks and Turk among Germans’.), embodied difference that a feeling is recognized in some contexts and marked as outsiders in others (I.9: ‘My appearance from outside is Turkish, my inner is hundred percent German’.).

For some, the threshold was experienced through repeated exclusion. Participant I.15, who was born and raised in Germany, described Turkey as her hometown while emphasizing that in Germany ‘you are always a foreigner’. Participant I.13 linked in‐betweenness to divided attachments and attenuated social relations:

We are stuck in‐between. We miss the events in Turkey when we are here. Same, when we come back. We couldn't get used to it. We have connections (in Germany) through work, we meet with colleagues, chat, and share our ideas. Still, it is not like living in one society. (I.13)

Category inclusiveness was therefore negotiated through movement between social worlds rather than settled by formal citizenship. The election made these shifting boundaries politically salient by asking participants which community could legitimately claim them and in whose name they might act.

### Category norms: Who should vote, and under what conditions

Participants articulated competing rules for legitimate transnational voting. Some treated voting as an unconditional entitlement attached to citizenship and therefore portable across borders:

It is absolutely wrong to say you cannot vote in Turkey. If we are Turks, we can vote there. If we obtain German citizenship, we can vote here, too, because we have our rights. There is no such thing as you cannot vote here, you cannot vote there. You cannot simply give up these rights because someone wants you to. (I.11)

Others tied political participation to residence and exposure to the consequences of government decisions. Participant I.16 argued that people should vote where they live because non‐residents lack everyday knowledge of conditions in Turkey: ‘I live here. I don't know the situation in Turkey. We should not nose into’ (I.16). Still others suspended judgement because they felt insufficiently informed about Turkish parties or uncertain about future consequences: ‘I wouldn't vote for the AKP… I don't know whom I would vote for… people say CHP, but I don't know how that would turn out either’ (I.8).

These normative positions were embedded in wider experiences of deservingness, loyalty and exclusion. Some long‐settled participants distinguished themselves from more recent arrivals, seeking recognition for their own belonging while reproducing boundaries against ‘newcomers’.

### Category interests: What voting is expected to achieve

Category interests concerned what participation was expected to accomplish for a relevant group and which future it was meant to make possible. Some participants understood voting as a means of asserting presence, demonstrating collective strength, protecting rights or preserving an alternative place of belonging under conditions of anti‐immigrant and anti‐Muslim hostility. In this reasoning, electoral efficacy did not depend solely on winning. Participation could also communicate that a minoritized collectivity existed and was capable of coordinated action.

One participant described asking her children to vote for DAVA (pro‐Erdogan alliance constructed in Germany) in the 2024 European elections, not because she expected the organization to win, but to show the power amid fears of deportation and civil conflict. Her account connected memories of exclusion, present insecurity and an imagined future in which Turkey might become a necessary refuge:

Our fears have increased. A civil war may break out, we do not know. Even police and officers who are xenophobic can tolerate their actions… I told my children to vote for DAVA. They cannot win, but at least show our power… Think about it. If they deport us in the future when there is a civil war, everybody can only flee to their home country. (I.15)

Memories of racist violence and anxiety about the rise of the German right also shaped participants' judgements about political membership and security.

They are (AfD supporters) racist. It is worrying. One becomes afraid. I am stressed because we know their past. Will they resurrect? I get nervous when I hear a crackle. I can't leave my window open even it is hot. They set people's houses on fire. (I.5)

Other participants managed this uncertainty by locating the threat of right‐wing extremist elsewhere, or placing trust in constitutional institutions: ‘It is worrisome, but mostly in the East. The constitutional state would not let them take the power’ (I.14). I.11 similarly withheld judgement about the AfD because political rhetoric and action might diverge in the future: ‘I cannot think of anything because I don't know what it (AfD) is going to do. It is politics, saying and doing are not always the same. So, we need to see’ (I.11). Norms of transnational voting were thus negotiated within a threshold between competing political contexts: rights in Turkey were evaluated through residence in Germany, while insecurity in Germany could heighten the perceived importance of maintaining political options elsewhere.

This account illustrates how the post‐liminal meaning of voting extended beyond the 2023 Turkish election. The electoral occasion became part of a longer chain of political moments through which participants assessed insecurity, group efficacy and future options across borders. Voting could therefore function as protective transnationalism, but this response was uneven. The same in‐between position also generated ambivalence, scepticism and withdrawal when participants doubted their knowledge, entitlement or capacity to influence either political context.

## DISCUSSION

Study 2 shows that participants did not merely reproduce frames supplied by parties, associations or public debates. They interpreted transnational voting through experiences of racism, memories of violence, rights‐based claims, uncertain futures and plural identifications. SAGA clarifies how these interpretations took the form of group arguments about category inclusiveness, category norms and category interests (Reicher & Sani, 1998; Sani & Reicher, 1999). The relevant ‘we’ was continually redrawn as Turkish, German, Kurdish, Muslim, hyphenated or positioned between Germany and Turkey; it was not fixed in advance by origin or citizenship (Cárdenas et al., 2021; Kahn, 2024; Sayad, 2004; see also Thomassen, 2014).

The interviews also demonstrate that liminality concerns more than occupying two national spaces. It is relational and processual (see Stenner, 2017). Participants drew past experiences of migration and racist violence into the present while anticipating democratic change, exclusion, deportation or return. From their post‐election perspective, the 2023 vote remained a reference point through which continuity and possible transformation were evaluated. Yet no single outcome followed from this threshold position. It could sustain entitlement claims and collective mobilization but also justify residence‐based restrictions, uncertainty or non‐participation.

Competing norms of transnational voting crystallized this tension. For some, citizenship created an enduring right to participate regardless of residence; for others, legitimate influence required everyday exposure to the consequences of political decisions. The question of who should vote therefore became a means of negotiating responsibility, loyalty, knowledge and membership across borders, echoing wider debates on external voting and transnational citizenship (Bauböck, 2016; Burgess, 2020; Caramani & Grotz, 2016b; Lafleur, 2013; Owen, 2011).

Category interests explain when this contested occasion became meaningful collective action. Voting was expected by some participants to protect rights, maintain symbolic presence, demonstrate efficacy or preserve future options under perceived insecurity in Germany. It was thus not reducible to homeland loyalty; it could operate as protective transnationalism. Read alongside Study 1, these findings show how organizationally staged meanings were accepted, modified, rejected or redirected in everyday reasoning. Transnational voting becomes collective action when infrastructures of mobilization intersect with participants' own constructions of who ‘we’ are, how ‘we’ may legitimately act and which collective future action is meant to secure (Klandermans, 1997, 2004; Simon & Klandermans, 2001; van Zomeren et al., 2008).

## GENERAL DISCUSSION

This article examined transnational voting as collective action among Turkey‐origin residents in Germany under conditions of dual exclusion in a liminal occasion. The two studies shed light on different but interdependent dimensions of this process. Study 1 showed how political alliances transformed formal voting rights into collective action by providing organizational infrastructures, trusted networks, symbolically charged venues and ideological repertoires. Study 2 showed that these mobilizing efforts did not determine participation in any straightforward way. Participants actively interpreted, accepted, modified, rejected or reworked organizational frames as they negotiated group boundaries, the legitimacy of transnational participation and the collective interests that voting was understood to serve.

Research on voting has typically treated it as a fixed and stable behavioural output, captured in binary terms—voted or not voted, or a choice among candidates or parties. This framing implicitly positions voting as a discrete act rather than a process, and says little about what precedes, accompanies or follows it. Approaching the 2023 elections as a liminal occasion offers a different starting point. It treats voting not as an isolated behavioural outcome but as a passage embedded in organizational mobilization, contested group boundaries and competing narratives about past and future—the conditions under which collective action theory suggests political participation becomes organized and consequential. This reframing makes it possible to ask not only whether someone voted, but how that act was organized, for whom it was made meaningful and what collective stakes it was understood to carry.

Building on this reframing, a central contribution of the study to social and political psychology of collective action is to conceptualize liminality as a process that shapes the collective action of those who may be simultaneously experiencing inclusion to some extent yet incomplete recognition within society. This ambiguous position makes questions of belonging, legitimacy and political responsibility especially salient. Transnational voting, therefore, becomes a particularly charged site of collective identification and collective action in plurality: participation provides an opportunity to define who belongs to the political community, whose interests should be represented and what obligations follow from membership from different social categories and political promises. Voting may express solidarity, loyalty, opposition, protection, continuity or demands for recognition, whereas abstention may reflect principled objections to transnational political authority or rejection of available collective categories.

The broader theoretical contribution of the present study is to bring collective action theory, liminality and the social identity approach into closer dialogue (Klandermans, 1997, 2004; Reicher & Sani, 1998; Sani & Reicher, 1999). Collective action perspectives illuminate how organizations construct grievances, efficacy, identities and benefits; liminality highlights the instability of membership and legitimacy in transnational settings by drawing attention to how actors themselves argue about collective identities and interests. Taken together, the findings suggest that collective action is not only enabled by pre‐existing group identities. It can also be an arena in which the group itself—its boundaries, obligations, legitimate representatives and shared interests—is actively constructed and contested.

This study is not free from limitations. Study 2 draws on a comparatively small number of interviews, which limits the range of positions and experiences that could be captured within an already heterogeneous population. The interviews were also conducted approximately 14–15 months after the 2023 elections, in a single German city; participants' accounts therefore reflect a retrospective, post‐liminal perspective shaped by the intervening period rather than the immediate electoral experience, and cannot speak to how transnational voting is interpreted beyond that one setting. Study 1 is similarly restricted to a single electoral cycle, the 2023 Turkish national elections, observed in three unnamed mid‐sized German cities, and cannot speak to how the organizational staging of transnational voting varies across elections, over time or in other local contexts.

Future research should therefore examine non‐participation and ambivalent participation more closely, compare other migrant populations and settlement contexts, and investigate how liminal political positions change over time. Longitudinal research would be particularly valuable for determining whether liminality is reproduced, intensified or partially resolved through naturalization, generational change, changing electoral institutions and transformations in origin‐ and settlement‐country politics. Further work should also examine how citizenship status, generation, class, ethnicity, religion, gender and political orientation intersect in shaping access to organizations and the legitimacy attributed to transnational participation. Under conditions of rising nativism and anti‐immigrant politics, transnational voting may become increasingly important as both a resource for collective agency and a site at which migrants' belonging is questioned. Its significance lies precisely in this tension: it can provide recognition and political voice while simultaneously exposing the unsettled and contested character of membership across borders.

## AUTHOR CONTRIBUTIONS

**Meral Gezici Yalçın:** Conceptualization; investigation; writing – original draft; methodology; visualization; formal analysis; project administration; data curation. **Elif Sandal‐Önal:** Conceptualization; methodology; investigation; writing – review and editing; formal analysis; data curation.

## CONFLICT OF INTEREST STATEMENT

The authors declare no competing interests.

## Footnotes

^1^
In the 2023 Turkish national elections, political parties competed largely through electoral alliances. The People's Alliance (Cumhur İttifakı) was led by the Justice and Development Party (AKP) and included the Nationalist Movement Party (MHP), the Great Unity Party (BBP), and the New Welfare Party (YRP). The Nation Alliance (Millet İttifakı) was composed of the Republican People's Party (CHP), the Good Party (İYİ Party), the Felicity Party (SP), the Democracy and Progress Party (DEVA), the Future Party (GP), and the Democrat Party (DP). The Labour and Freedom Alliance (Emek ve Özgürlük İttifakı) brought together the Peoples' Equality and Democracy Party (Green Left Party/Yeşil Sol Parti during the election period, later DEM Party), the Workers' Party of Turkey (TİP), the Labour Party (EMEP), the Labourist Movement Party (EHP), the Social Freedom Party (TÖP), and the Federation of Socialist Assemblies (SMF). Throughout this manuscript, references to these alliances correspond to these electoral coalitions as they existed during the 2023 elections.
